# Birth, life and death of nascent polypeptide chains

**DOI:** 10.1002/biot.201000327

**Published:** 2011-06

**Authors:** Sujata Jha, Anton A Komar

**Affiliations:** Center for Gene Regulation in Health and Disease, Department of Biological, Geological and Environmental Sciences, Cleveland State UniversityCleveland, OH, USA

**Keywords:** Co-translational protein folding, Co-translational protein modifications, Kinetics of translation, Nascent polypeptide chain, Synonymous codon usage

## Abstract

The journey of nascent polypeptides from synthesis at the peptidyl transferase center of the ribosome (“birth”) to full function (“maturity”) involves multiple interactions, constraints, modifications and folding events. Each step of this journey impacts the ultimate expression level and functional capacity of the translated protein. It has become clear that the kinetics of protein translation is predominantly modulated by synonymous codon usage along the mRNA, and that this provides an active mechanism for coordinating the synthesis, maturation and folding of nascent polypeptides. Multiple quality control systems ensure that proteins achieve their native, functional form. Unproductive co-translational folding intermediates that arise during protein synthesis may undergo enhanced interaction with components of these systems, such as chaperones, and/or be subjects of co-translational degradation (“death”). This review provides an overview of our current understanding of the complex co-translational events that accompany the synthesis, maturation, folding and degradation of nascent polypeptide chains.

## 1 Introduction

Protein folding is an intricate process. Proteins must achieve their native conformation to be fully functional. Therefore, it is essential to understand the exact mechanism of protein folding and the precise effects of various factors/conditions that could impact the process. Our current understanding of protein folding is predominantly based on in vitro denaturation/renaturation experiments involving (mostly) purified natural and/or recombinant full-length proteins and/or in silico modeling studies. The seminal denaturation/renaturation experiments performed by Christian Anfinsen and his colleagues in the 1950–60s indicated that the folding code is contained entirely in the amino acid sequence of the protein [1]. Computer-based simulation experiments seemed to corroborate this conclusion [2, 3]. Thus, the primary sequence of the polypeptide is believed to contain both necessary and sufficient information to specify its unique 3D structure [1–3]. However, despite obvious progress in the field and the availability of new technologies and powerful computers, the protein folding code still remains undeciphered.

A comprehensive understanding of protein folding requires elucidation of the protein folding mechanism under native intracellular conditions. In vivo protein folding occurs in a crowded cellular environment and is widely believed to start during protein synthesis on the ribosome, i.e., co-translationally [4–6]. Co-translational folding is a vectorial process, proceeding step-wise from the N terminus to the C terminus of the nascent polypeptide chain as it emerges from the ribosome [4–6]. During this process the nascent polypeptide remains tethered to the ribosome through its C-terminal end. It has become clear that numerous events accompany protein synthesis and folding on the ribosome [4–6]. After its birth at the peptidyl transferase center (PTC) of the ribosome, the nascent polypeptide begins its journey “from youth to maturity” through the ribosomal tunnel to the cytosol to become a functional protein. Almost immediately after its birth at the PTC, the nascent chain is subjected to multiple interactions, constraints and folding events [4–6]. The exit of the nascent chain out of the ribosome tunnel is often coupled to various modifications and interactions with export targeting particles (i.e., signal recognition particle), chaperones and/or folding catalysts [4–6]. The rate of protein synthesis is (mostly) modulated by codon usage in the messenger RNA (mRNA) being translated, and this actively facilitates the co-translational folding process and channels it to the most productive pathway [4]. The in vivo protein-folding process, however, is not 100% efficient. Unproductive co-translational folding intermediates also form during protein synthesis. Such aberrant intermediates may undergo enhanced interaction with quality control systems (e.g., chaperones) and/or be subjects of co-translational degradation [7, 8].

**Figure 1:**
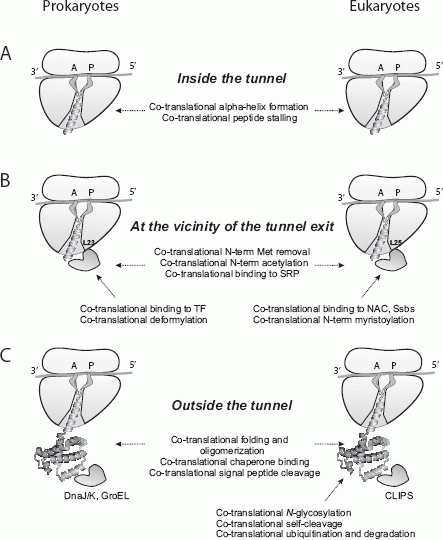
Schematic representation of the complex co-translational events accompanying synthesis and biogenesis of nascent polypeptide chains in pro- and eukaryotes. (A) Events accompanying nascent peptide growth inside the ribosomal tunnel: co-translational α-helix formation and co-translational peptide stalling occur in both pro- and eukaryotes. (B) Events accompanying nascent peptide growth and maturation in the immediate vicinity of the tunnel exit: co-translational N-terminal Met removal, co-translational N-terminal acetylation and co-translational binding to signal recognition particle (SRP) occur in both pro- and eukaryotes; co-translational binding to trigger factor (TF) and co-translational deformylation are specific to prokaryotes; co-translational binding to nascent associated complex (NAC), Ssbs and co-translational N-terminal myristoylation are specific for eukaryotes. (C) Events accompanying nascent peptide growth and maturation outside the ribosomal tunnel: co-translational folding and oligomerization, co-translational chaperone binding and co-translational signal peptide cleavage occur in both pro- and eukaryotes; co-translational *N*-glycosylation, co-translational peptide self-cleavage, co-translational ubiquitination and degradation are specific for eukaryotes.

In this review, we provide an overview of the complex co-translational events that accompany synthesis, maturation, folding and degradation of nascent polypeptide chains (Fig. 1).

## 2 Birth of polypeptides via peptide bond formation at the ribosome PTC

The ribosome is a large nucleoprotein complex that translates the information contained in an mRNA to produce the encoded protein [9]. Ribosomes from all organisms are composed of two subunits. The small subunit contains the decoding center that directs pairing of the appropriate aminoacyl-transfer RNAs (aa-tRNAs) with triplets (codons) in the mRNA. The large subunit harbors the catalytic (peptidyl transferase) center that joins the amino acids together with peptide bonds [9, 10]. In all living organisms the translation process involves four basic steps: initiation, elongation, termination and ribosome recycling [9–12]. Of these steps, elongation is the most predominant as it accounts for all but one of the amino acids in the completed polypeptide chains [13, 14]. During the initiation step of protein synthesis the ribosome is positioned at the AUG codon of the mRNA with the initiator tRNA (Met-tRNA_i_) in the peptidyl or P site of the ribosome [13, 14]. This placement sets the reading frame for all subsequent aa-tRNAs coming into the A site of the ribosome. The Genetic Code dictates which aminoacyl-tRNA will then bind to the A site during the decoding process regulated by the small ribosomal subunit. Decoding dictated by triplets (codons) in the mRNA is followed by peptide bond formation catalyzed by the PTC in the large ribosomal subunit. Translation elongation involves sequential addition of amino acids to the polypeptide chain and is facilitated by a set of extremely conserved elongation factors (for detailed reviews see [13, 14]).

X-ray structures of the prokaryotic (70S) ribosome provided detailed information about the structure of the PTC and the mechanism of peptide bond formation [9, 10]. In addition, valuable structural information has been obtained from cryoelectron microscopy (cryo-EM) structures of ribosomes of both prokaryotic and eukaryotic origin [15, 16]. Recent progress in solving the X-ray structures of the eukaryotic yeast (*Saccharomyces cerevisiae*) 80S ribosome [17] and the 40S ribosomal subunit of the ciliate protozoan *Tetrahymena thermophila* [18], as well as determination of the cryo-EM structures of a plant (*Triticum aestivum*) 80S ribosome, further advances our knowledge in the field [19, 20].

It is believed that the general mechanism of peptide bond formation is the same in all kingdoms of life. The central event in this process is the nucleophilic attack of the amino group of the amino acid in the A site on the ester carbon of the amino acid of the peptidyl-tRNA in the P site [9, 10]. The decoding center itself appears to be devoid of proteins [9, 10], suggesting that ribosomal RNA (rRNA) directs peptide bond formation, while both rRNAs and the ribosomal proteins serve as a scaffold for proper positioning of the substrates. It was proposed that a reactive N3 amine of the conserved nucleotide A2451 in the rRNA was critical for the general mechanism of peptide bond formation; however, this was disproved by recent evidence (for a review see [9]). Thus, to date, the exact details of the catalytic mechanism of the PTC remain unknown.

## 3 The ribosome tunnel

After synthesis at the PTC, polypeptides are believed to begin traveling through the ribosomal exit tunnel (or “protein conducting channel”) in the large ribosomal subunit [21–24]. Detailed insights into the architecture of the tunnel have been obtained from X-ray and cryo-EM structures of prokaryotic and eukaryotic ribosomes (for a review see [24]). The tunnel is ∼80–100 Å long and its diameter varies from ∼10 Å at its narrowest region to ∼20 Å at its widest region (at the exit site) [24]. The tunnel is expected to cover ∼30–40 amino acids of the elongating polypeptide at a time [21–30] (assuming that the polypeptide is in a fully extended conformation).

While it is widely believed that all nascent chains pass through the ribosome tunnel, it remains unclear whether this is a strictly true. Some studies employing, for example, the hot tritium ([^3^H])-bombardment technique [31] and/or immune electron microscopy [32] detected very short nascent peptides (from 2 to 42 amino acids in length) exposed on the surface of ribosomes [31, 32]. However, comparative cross-linking experiments using *Escherichia coli* ribosomes showed that with increasing length, nascent peptides become predominantly and progressively cross-linked to sites within domains V, IV, II, III and I of the 23S rRNA (which form the ribosome tunnel entrance and walls, respectively). These cross-linking results further suggested that the peptides do pass through the tunnel [33–36]. Moreover, occasional exposure of short peptides on the surface of ribosomes was proposed to be due to abortion of translation and subsequent attachment of the released incomplete chains to the ribosome [33–36]. To visualize the nascent chain inside the tunnel one would require a structure with at least 3.5-Å resolution, which is not yet available for ribosome-bound nascent chain complexes. However, recent cryo-EM reconstructions (done at ∼6–7-Å resolution) of ribosomes containing nascent chains [24, 37–39] provided intriguing details of the complex interactions between the nascent chain and the ribosome (see below).

The ribosome tunnel wall is composed primarily of the negatively charged rRNA (23S in prokaryotes and 28S in eukaryotes) and the ribosomal proteins L4, L22 and L23 in prokaryotes and L4, L17 and L25 in eukaryotes [5, 18, 24]. It should be noted, however, that protein contributions to the formation of the tunnel are thought to be limited to the narrow “constriction” region of about 10–20 Å in length (∼30 Å away from the PTC) and to a ∼20–30-Å-long “lower” region at the exit (“vestibule”) site of the tunnel ([24] and references therein). The constriction region of the tunnel is predominantly formed by the protruding loops of the L4 and L22 (L17) proteins, whereas the exit site is largely comprised of the L23 (L25) protein [5, 18, 24]. The electrostatic potential in the tunnel is mostly negative [40].

For a long time, it was widely believed that the ribosomal tunnel merely acts as an inert passageway for nascent polypeptide chains [25–28]. However, emerging evidence indicates that the ribosomal tunnel is not a passive conduit, but rather may actively participate in nascent chain folding [29, 30, 40], and in the regulation of protein synthesis via modulation of PTC activity [41–44]. In this regard, it is notable that a number of leader peptides have been shown to induce translational stalling in response to cellular stress conditions and/or presence or absence of effector molecules, thereby providing a means to regulate expression of downstream genes [41–44]. Bacterial SecM, TnaC, RrmC and MifM are the best known examples of such regulatory proteins/peptides [41–43]. Below, we discuss two representative cases (SecM and TnaC).

### 3.1 Stalling of translation inside the ribosome tunnel induced by nascent SecM and TnaC peptides

SecM (secretion monitor) is a 170-amino acid *E. coli* protein that regulates expression of the downstream SecA (secretion driving) ATPase in the *secM-secA* operon [43, 45]. SecA is a central component of the translocase in *E. coli*, which drives the transmembrane movement of the pre-protein and its insertion into integral membrane complex, secYEG, the translocon [46]. Expression of secA is controlled by specific mechanisms that respond to the protein secretion status of the cell [43, 45]. SecA expression is elevated under conditions of compromised translocase activity [43, 45]. SecM was shown to play an important role in this event [43, 45]. SecM is itself a periplasmic protein and, under normal conditions, is rapidly degraded after its export to the periplasm [43, 45]. However, SecM translation was shown to be subject to regulation via elongation arrest, which becomes prolonged when export of nascent SecM is blocked (e.g., due to compromised translocase activity) [43, 45, 47]. Thus, SecM regulates its own co-translational export.

Interestingly, SecM translational arrest was shown to facilitate *secA* expression [43, 45, 47]. A 17-amino acid sequence (150-FSTPVWISQAQGI-RA**G**P-166) in the C-terminal region of SecM was found to cause stalling of SecM elongation at Gly165, thereby producing peptidyl-glycyl-tRNA bound to the P site [43, 45, 47]. The next codon (encoding Pro166) was essential for arrest, suggesting that prolyl-tRNA acts as an A-site effector of this event [43, 45]. Translational stalling in this case is thought to promote remodeling of the *secM-secA* mRNA at the intergenic region to expose the Shine-Dalgarno sequence preceding the *secA* open reading frame (ORF), and enhance recruitment of 30S ribosomal subunits and initiation of translation of the SecA protein [43, 45]. Nine residues within the SecM arrest sequence (shown in bold type; 150-**F**XXXX**WI**XXXX**GIRAGP**-166) were shown to be of major importance in modulating the efficiency of SecM translational stalling [47].

The role of the ribosomal tunnel in the SecM stalling process was highlighted by mutational analysis of the L4 and L22 proteins and the 23S rRNA [24, 47, 48]. A number of specific mutations in the protruding loops of L4 and L22 and in the 23S rRNA (e.g., 23S rRNA nucleotides A749–A753 and A2058 at the constriction site and nucleotides A2503 and A2062 at the tunnel entrance) were shown to abolish nascent peptide-dependent stalling [47, 48]. These results indicate an active contribution of the components of the ribosome tunnel during stalling.

Another example of translational regulation via nascent peptide-mediated stalling is that controlled by the 24-amino acid *E. coli* TnaC regulatory peptide [38, 42, 43, 49]. The TnaC peptide is produced from the *tnaC* ORF located upstream of the tryptophanase (*tnaA*) gene in the *tna* operon of *E. coli* and regulates tryptophanase expression in response to tryptophan levels in the cell [49]. When levels of free tryptophan are low, the TnaC peptide is properly translated and terminated. This results in dissociation of the mRNA and ribosomes, which allows the Rho factor to access the transcription termination site and terminate transcription before the RNA polymerase reaches the downstream tryptophan-catabolizing genes in the *tna* operon. Elevated tryptophan levels lead to ribosome stalling mediated by TnaC. The stalled complex masks the Rho-dependent transcription termination site, thus allowing the transcription of the downstream tryptophan-catabolizing genes to proceed [49]. As in the case of SecM, a number of residues in the TnaC peptide (including Trp12, Asp16 and Pro24 [49–51]) and in components of the ribosomal tunnel (L22 protein and 23S rRNA) were shown to be critical for the stalling process [38, 49–51]. However, it appears that the ribosome may use different mechanisms to recognize different stalling peptides [24, 48, 52]. For example, TnaC translational arrest (in contrast to SecM arrest) was found to be insensitive to A2503 or A2062 mutations [48].

One of the interesting and yet unresolved questions in this process is the impact of nascent peptide compaction (i.e., folding) inside the ribosome tunnel on the stalling mechanism. Fluorescence resonance energy transfer (FRET) experiments suggested that the nascent SecM peptide might (at least in part) acquire a compact α-helical structure within the tunnel [53]. In contrast, cryo-EM analysis of the 70S ribosome-TnaC complex indicated that, when inside the tunnel, the TnaC peptide is predominantly in an extended conformation, although some compaction of the TnaC peptide in the vestibule region of the tunnel could not be also excluded [38].

While it is clear that some peptides can direct translational stalling, the exact nature and mechanism of signal transmission from the stalled peptide to the PTC resulting in translational arrest is unknown. Cryo-EM of the 70S·TnaC complex at 5.8-Å resolution suggested that communication between the stalled peptide and the PTC does not involve substantial conformational changes of the tunnel itself [38]. It was proposed that the “stalling signal” might propagate back to the PTC through the nascent chain, via induction of a specific conformation within the chain that inhibits the PTC [53, 54]. Recent evidence indicates that the stalling sequence(s) may cause impairment of the PTC A site by changing its conformation, thereby triggering elongation arrest [44]. These possibilities suggest that the ribosome tunnel might have evolved to be capable of sensing not only specific amino acid residues and their location within the nascent peptide, but also specific conformations of nascent chains, all of which together dictate the translational fate of the encoded (downstream) protein(s) in response to, e.g., environmental cues [24].

Translational control via nascent peptide-mediated stalling is not limited to bacterial systems. For example, the translation of mRNAs without a stop codon harboring poly(A) tail in yeast is considered as an eukaryotic example of nascent peptide-induced translational arrest caused by a poly(A)-encoded polylysine stretch at the C terminus of the proteins, which causes subsequent degradation of such aberrant proteins by proteasome [55, 56]. The *Neurospora crassa* arg-2 uORF encoding a 24-residue arginine attenuator peptide is another example of an eukaryotic arrest sequence. This peptide directs stalling of ribosomes at the uORF stop codon in response to arginine, thereby blocking ribosomes from reaching the ARG-2 initiation codon under conditions of sufficient arginine [57]. ARG-2 encodes arginine-specific carbamoyl phosphate synthetase, the first enzyme in fungal arginine biosynthesis. A number of residues within the arg-2 uORF (Asp12,Tyr13, Lys14, and Trp19) have been identified as critically important for the stalling process [57].

In summary, studies of the stalling sequences of SecM, TnaC and other peptides suggest that the nature of the amino acid residues passing through the tunnel, the distance of these critical residues from the PTC, their interaction with the tunnel walls, and perhaps the conformation of the peptide, may all play important roles in the regulation of nascent peptide-mediated translational arrest [24]. The nascent peptide sequences that have been identified as directing stalling are portable and are useful tools for studying co-translational protein folding [6]. Nevertheless, the precise molecular mechanisms involved in recognition of the critical nascent peptide sequences and in the execution of ribosome stalling have yet to be determined. In addition, the nature and extent of interactions between the tunnel interior and non-stalling nascent peptide sequences passing through the tunnel remain unclear.

### 3.2 Co-translational folding inside the ribosome tunnel

From cryo-EM studies visualizing stalled peptides inside the ribosome tunnel [24], it is evident that the elongating peptide starts to fold very early in its genesis, perhaps immediately at the PTC (Fig. 1A). The first theoretical presentation of such a possibility was made by Lim and Spirin in the mid- 1980s [58]. Stereochemical analysis of the transpeptidation reaction allowed them to suggest that the ribosome likely generates an α-helical conformation at the C-terminal end of the nascent peptide [58]. The groups of Arthur Johnson and Carol Deutsch experimentally verified this hypothesis using FRET and/or a combination of accessibility assays, respectively [29, 30, 53, 59]. There is now ample evidence indicating that peptides can acquire compact (α-helical) structures in the ribosomal exit tunnel near the PTC and in the lower ∼20–30-Å “vestibule” region of the tunnel [24, 30, 39, 53, 59, 60]. Moreover, it has been suggested that the tunnel may actively modulate peptide secondary structure formation via induction and stabilization of α-helices within regions of the nascent chain with high helix-forming propensity and deferring regions lacking such propensity from helix formation [53, 60]. It remains an open question, however, to what extent the tunnel itself may change in conformation, and whether peptide sequence(s) with strong helix-forming propensity might be capable of modulating the width of the tunnel to allow the corresponding polypeptide stretches to acquire secondary structure all along the tunnel [60]. It has been suggested that the “vestibule” (lower) region of the tunnel may be structurally dynamic and capable of accommodating even higher order (tertiary) structures [61–63].

## 4 Interactions and modifications of the nascent polypeptide chain outside the tunnel

The fate of the nascent polypeptide chain (including its conformation) outside the ribosome tunnel is to a substantial extent governed by the ultimate destination of the protein within the cell (its intra-cellular compartmentalization and/or secretion) as well as its modifications. Upon extrusion out of the tunnel, nascent chains are subjected to subsequent folding events and interactions with chaperones and modifying enzymes [5]. As discussed below, many of these events are thought to take place in the vicinity of the tunnel exit since many nascent chain-interacting proteins and enzymes appear to be associated with the ribosome [5]. Many of the studies devoted to analysis of nascent chain conformation inside the tunnel utilized either model/fusion sequences and/or fragments of transmembrane/secretory proteins, such as fragments of the vesicular stomatitis virus (VSV) G glycoprotein and/or voltage-gated potassium (Kv) channel protein [29, 30, 40, 59, 60, 62]. Segments of the VSV G glycoprotein that were shown to form α-helices inside the tunnel were found to retain their compact helical structures during their subsequent movement through the translocon [59]. Thus, once formed, 2-D helical structures within transmembrane protein(s) appeared to be retained during their journey through both the tunnel and the translocon, thereby facilitating their integration into the membrane [59].

It should be noted that in both prokaryotes and eukaryotes, entry of secreted and membrane-bound proteins into the translocon involves interaction of the proteins' signal peptide with signal recognition particle (SRP). This is discussed further below.

### 4.1 Interaction of nascent chains with SRP

Proper sorting and compartmentalization of proteins is essential for their functions and for overall cellular function. For secreted proteins and the majority of the membrane-bound proteins, this involves interaction of the SRP with (mostly) hydrophobic signal sequences (see below) within the nascent chains as they emerge from the ribosome. SRP targets nascent polypeptides to the membrane-associated translocation machinery through association with its membrane receptor(s), thereby facilitating subsequent export and/or membrane integration of the polypeptide [64–66].

SRP is a universally conserved ribonucleoprotein (RNA-protein complex); however, the size and structure of the RNA and the number of proteins involved in SRP formation varies [64–66]. In prokaryotes, SRP is composed of a single protein (Ffh) and a small RNA (4.5S). In eukaryotes, SRP contains six distinct proteins (including SRP54, which is homologous to Ffh) and a 7S RNA (one domain of which shares sequence and structural homology with the prokaryotic 4.5S RNA) [64–66].

Biochemical and structural studies have identified the ribosomal protein L23 (L25 in eukaryotes) as the main determinant of ribosome-SRP interaction [65, 66]. This interaction seems to occur independently of the nascent peptide in the exit tunnel of the ribosome [67]; however, it accelerates targeting of the ribosome nascent chain complex to the translocon [64–66]. The signal peptide is believed to interact first with the ribosomal proteins L4 and L22 inside the tunnel, then with L23 at the exit site of the tunnel, and subsequently with SRP [65]. A recent crystal structure of the Ffh protein bound to a signal sequence revealed a putative induced-fit mechanism that leads to accommodation of the signal sequence within the hydrophobic groove of the SRP protein [68]. Interaction between SRP and the signal sequence may (as in eukaryotes), or may not (as in bacteria) temporarily arrest translation of the nascent chain containing the signal sequence [64–66].

Following its assembly, the ribosome-nascent chain-SRP complex is targeted to specific SRP receptors (e.g., FtsY in *E. coli*, which is homologous to the α-subunit of the eukaryotic receptor), which facilitates subsequent assembly of the translocon. A recent cryo-EM structure of the *E. coli* ribosome in complex with SRP and the FtsY receptor demonstrated that the ribosome acts as a platform that optimally positions critical SRP regions for receptor interaction [69]. The Ffh component of SRP and the FtsY SRP receptor are both GTPases and bind GTP before complex formation [64–66]. Multiple distinct conformations of the SRP-FtsY complex have been identified that orchestrate the transfer of the ribosome-bound SRP complexes to the translocon [70]. An early, GTP-independent complex subsequently rearranges to an “activated state” complex. Upon GTP hydrolysis, the SRP complex dissociates and co-translational translocation of the nascent chain proceeds [70].

We would like to emphasize that this is a simplified overview of the complex events underlying co-translational translocation of proteins, and that many aspects of these processes remain to be elucidated in both prokaryotes and eukaryotes.

Cytosolic nascent chains that do not cross membranes during their maturation and do not interact with SRP seem to differ in at least certain aspects of their “life” as compared to secreted and membrane-bound polypeptides. Cytosolic nascent chains might be expected to start folding into higher order 3-D structures almost immediately after leaving the “vestibule” exit region of the ribosome tunnel (see below). However, at least some of cytosolic nascent chains have been shown to first interact with ribosome-associated chaperones and/or other ribosome-associated protein complexes and enzymes [5, 71]. Many of these interactions and consequent events are actually shared between secreted/transmembrane and cytosolic proteins [5, 71]. Representative examples are discussed below.

### 4.2 The prokaryotic ribosome-associated chaperone, trigger factor

Trigger factor (TF) is the only ribosome-associated chaperone indentified to date in bacteria [72]. *E. coli* TF is a constitutively expressed, ATP-independent and abundant chaperone [72]. TF has two major activities, functioning as a peptidyl-prolyl *cis/trans* isomerase (PPIase) and as a chaperone that aids in proper folding of nascent polypeptides [72]. Work showing that TF lacking the entire PPI-ase domain maintains its chaperone activity in vivo demonstrated that the PPIase activity of TF is not required for its chaperone function [73]. TF exists in the cytosol mostly as a dimer, yet interacts with the ribosome predominantly as a monomer (via binding to ribosomal protein L23 at the exit site of the tunnel) [74]. Ribosomal protein L29 also participates in bridging interactions between the ribosome and TF [72, 74]. The X-ray crystallographic structure of TF has been solved in both its free and ribosome-bound forms [72, 75]. It should be mentioned that the affinity of TF for vacant ribosomes is rather low (∼1 μm), but is greatly increased (up to ∼10-fold) in the presence of a nascent polypeptide [72]. Interestingly, TF and SRP can simultaneously bind to ribosomes [72]. TF binding is abolished, however, when SRP binds to the SRP receptor FtsY [72]. TF has three domains, which form an elongated, “dragon”-shaped structure [72, 75]. The N-terminal domain (the “tail” of the structure) contains the signature “GFRXGXXP” motif, which is necessary and sufficient for interaction of TF with the ribosomal protein L23 [72, 75]. The middle domain (“head”) of TF is connected to the N-terminal domain via a long linker and carries the PPIase activity. The C-terminal domain is the largest domain and forms the central chaperone body of TF with two protruding “arms” on both sides.

Various X-ray and cryo-EM structures of TF bound to *E. coli* ribosomes are available and show the arch-like appearance of TF over the tunnel exit site [72, 75]. This architecture of the complex suggests that the position of TF over the tunnel might provide additional “protection” to nascent chains emerging out of the tunnel. TF was proposed to act as a cradle, aiding in nascent chain co-translational folding [72, 75]; however, the details of this mechanism remain unknown. Cross-linking experiments indicated that TF interacts with the nascent peptide using the entire interior surface of its arch [76]. This interior surface is mostly hydrophobic, with a few polar and charged residues, and is capable of interacting with a variety of nascent chains [72, 77]. TF binding is dependent on the size of the nascent chain as well as its sequence composition and structure (folding state) [72]. The interior space of the TF arch is of sufficient size to accommodate small globular protein fragments, suggesting that co-translational folding of globular domains might occur under the cover of the TF arch, before the nascent chain reaches the cytosol [72]. TF improves the yield of the correctly folded proteins, especially under stress conditions favoring misfolding [72]. However, since TF is not an essential protein [72], it is likely that only a fraction of emerging nascent polypeptides interact with TF. Nascent chains that do not require TF to achieve their proper conformation may do so in a chaperone-independent manner or utilize other ‘downstream’ chaperones (see further below).

### 4.3 Eukaryotic Hsp70s/ribosome-associated complex and the nascent associated complex

Unlike prokaryotes, eukaryotes have more than one ribosome-associated chaperone [5, 71]. These have been best characterized in the yeast *S. cerevisiae*. Yeast has two distinct ribosome-associated chaperone machineries: the Ssb/Ssz/Zuotin triad and the nascent associated complex (NAC) [78–81]. Both complexes are abundant and conserved among eukaryotes [78–81]. Two components of the Ssb/Ssz/Zuotin machinery are Hsp70 family members: Ssb (represented by Ssb1 and Ssb2) and Ssz. It also includes Zuotin (Zuo), the Hsp40 co-chaperone for Ssz [78–81]. Ssz and Zuo form a stable heterodimeric complex known as ribosome-associated complex (RAC) [80]. RAC acts as co-chaperone for Ssb and stimulates its ATPase activity via the N-terminal J domain of Zuo [80]. Ssz cannot bind ribosomes directly, while Ssb can (although the exact binding site remains unknown) [82]. Zuo bridges RAC to the ribosome via a charged region at the Zuo C terminus, which interacts with eukaryotic ribosomal protein L31, located in close proximity to the tunnel exit site [82].

The RAC complex is well conserved in mammals and consists of Hsp70L1 and Mpp11, homologs of Ssz and Zou, respectively [83, 84]. RAC and Ssb form part of the specific eukaryotic chaperone network termed “chaperones linked to protein synthesis” (CLIPS), which cooperates with the translational apparatus in assisting co-translational folding of proteins [85]. However, not all ribosome-associated CLIPS are capable of interacting with growing nascent chains. Neither Zuo1 nor Ssz1 can interact with nascent chains; thus, this property seems to be limited (in the triad) to Ssbs [85].

The second largest eukaryotic ribosome-associated chaperone complex is NAC. NAC is also well conserved in eukaryotes [81]. It is an ATP-independent heterodimeric protein complex composed of α and β subunits. Both the α and β subunits were shown to interact with nascent polypeptides; however, only the β subunit is involved in NAC interaction with the ribosome [81]. A conserved positively charged [RRK(X)nKK] ribosome-binding motif was identified within the β subunit of NAC that is essential for NAC complex attachment to the ribosomal protein L25 [81]. This N-terminal ribosome-binding domain is sufficient and necessary to target NAC to the ribosome. Mutations in the β subunit and in L25 reduce and/or abolish NAC binding to the ribosome in vivo and in vitro [81]. Therefore, ribosomal proteins L23 and L25 appear to function as universal docking sites for ribosome-associated factors and complexes seeking access to the nascent chains in prokaryotes and eukaryotes, respectively [5].

NAC was originally (and is still) considered to be the first eukaryotic nascent chain-interacting protein that binds elongating nascent polypeptides as they are being translated [86]. NAC was also suggested to shield nascent chains from the cytosol [87]. NAC depletion exposed very short nascent polypeptide chains (∼12 amino acids) to proteolysis [87], while under normal circumstances (NAC present), only longer nascent chains (more than 30–40 amino acids) were susceptible to proteolytic digestion on the ribosome [25, 26, 87]). Based on these findings, NAC was proposed to, at least in part, contribute to the formation of the tunnel walls [87], perhaps in the “vestibule” region. However, this hypothesis did not find any further experimental support. The observations that NAC associates with ribosomes and interacts with nascent chains led to the suggestion that NAC may actively participate in the folding of newly synthesized polypeptides [86, 87]. This also remains to be proven.

NAC is not essential in yeast; however, its deletion leads to embryonic lethality in mice, nematodes, and fruit flies [81]. It was proposed that NAC controls the co-translational targeting of nascent peptides to the endoplasmic reticulum (ER) by regulating the accessibility of the nascent chains to SRP [81]. Nevertheless, to a substantial extent, the role of NAC in eukaryotic translation remains enigmatic.

### 4.4 Other nascent chain interacting chaperones

It should be noted that interactions with nascent chains as they emerge out of the ribosome tunnel are not only limited to ribosome-associated chaperones and protein complexes such as those described above. Members of chaperone networks downstream of the ribosome-associated TF and/or Ssb-RAC/NAC complexes have also been shown to interact with nascent chains and facilitate their folding [5, 88]. In particular, members of both the Hsp70 (DnaK, DnaJ in bacteria) and Hsp60 (GroEL in bacteria) chaperone families were found to interact co-translationally with elongating nascent chains [5, 88, 89]. These types of interactions, however, are expected to occur on a “need-basis”, as in the case of the majority of proteins that undergo enhanced interaction with Hsp70/Hsp60 family members. Indeed, it appears that not only productive protein folding, but also misfolding, can occur co-translationally (see below).

Finally, in eukaryotes, nascent chains are also subject to co-translational interaction with enzymes of the protein disulfide isomerase family, which catalyze disulfide bond formation, reduction, and isomerization, as well as members of the quality control/calnexin chaperone system that is directed toward (Asn-linked glycosylated) secreted glycoproteins [90].

### 4.5 Co-translational protein modifications by ribosome-bound enzymes

The fate of nascent polypeptide chains is not only governed by their interaction with ribosome-associated ribonucleoprotein particles and nascent associated complexes and chaperones. Various protein modifications are known to affect protein folding, stability, activity, intracellular localization, and interaction with binding partners. Many of the enzymes involved in these modifications are associated with ribosomes (Fig. 1B) and are capable of acting on elongating nascent chains [5, 71]. We review a number of representative examples below.

#### 4.5.1 Co-translational deformylation of N-terminal formylmethionine

In eubacteria, mitochondria and chloroplasts, protein translation is initiated by a specialized transfer RNA charged with formylmethionine (tRNAf^Met^) [91]. Formylation of the N-terminal methionine blocks the reactive amino group and prevents unfavorable side reactions [91], thus enhancing the efficiency of translation initiation. Peptide deformylase (PDF) removes the N-terminal formyl group as soon as the nascent chain emerges from the ribosomal tunnel [92, 93]. It is the first ribosome-associated protein/enzyme that acts upon nascent chains in bacteria, mitochondria and chloroplasts [92–94]. PDFs are metalloproteases that have been shown to adopt a unique fold [92–94]. X-ray analysis of the complex between the positively charged PDF C-terminal helix (that recruits PDF to the ribosome) and the 70S *E. coli* ribosome suggested a model in which PDF and TF act synergistically to enable deformylation in the shielded environment provided by TF [93]. PDF is thought to bind in the groove between ribosomal proteins L22 and L32, with L22 serving as the major docking site [93]. It has been proposed that positioning of the PDF C-terminal helix in the groove orients the enzyme's active site toward the ribosomal tunnel exit [93]. PDFs are essential enzymes and their activities are indispensable for cellular function [92, 94].

#### 4.5.2 Removal of N-terminal methionine

Deformylation of the N-terminal methionine of nascent peptides is often followed in eubacteria by co-translational removal of the N-terminal methionine itself by methionine aminopeptidase (MetAP or MAP) [95, 96]. However, this process (also called N-terminal methionine excision, NME) is not limited to bacterial systems and is well conserved across all kingdoms of life [95, 96]. More than 50% of all cellular proteins are expected to undergo N-terminal methionine removal [95, 96]. Surveys of N-terminal sequences and in vitro experiments utilizing model peptide substrates have shown that the N-terminal methionine is removed if the following (second) residue is small and uncharged (e.g., Met, Gly, Ala, Ser,Thr, Pro,Val or Cys) [95, 96].

MAPs are ubiquitous and essential enzymes in all living organisms [95, 96]. Based on their domain structure, MAPs are classified as type I or type II [95, 96]. Eubacteria contain only type I MAPs, while eukaryotes contain both types. Type I enzymes have been further subdivided into type Ia and type Ib subclasses, depending on the presence or absence of an N-terminal extension that contains zinc finger motif(s) [95, 96]. This N-terminal extension was suggested (and experimentally proven in the case of yeast MAP1) to aid MAP recruitment to the ribosome [97, 98]. There are, however, relatively few data on the interaction between MAPs and ribosomes. Given their function, it is likely that MAPs (at least type Ia) bind to ribosomes in a way that positions the enzyme close to the ribosome tunnel exit.

#### 4.5.3 N^α^-terminal acetylation

Acetylation is the most common co-translational protein modification [99, 100]. Approximately 60% of yeast proteins and more than 80% of human polypeptides are acetylated ([99, 100] and references therein). This modification is, however, rather rare in bacteria and occurs less frequently in archea [99]. Acetylation occurs through N^α^-terminal acetyltransferase (Nat)-mediated transfer of the acetyl group from acetyl-CoA to the α-amino group of the protein's N-terminal amino acid [99, 100]. Eukaryotes contain three major Nats termed NatA, NatB, and NatC [100]. Each of the three Nats contains a catalytic subunit and one or two auxiliary subunits [100]. NatA is dependent upon the prior action of MAP and acetylates proteins beginning with Gly, Ala, Ser, Thr and sometimes Val and Cys [100]. The activities of NatB and NatC are independent of methionine removal by MAP. NatB is specific for N-terminal Met-Glu, Met-Asp and Met-Asn pairs [100]. NatC is specific for proteins starting with Met and having a bulky hydrophobic amino acid in the second position (Met-Ile, Met-Leu, Met-Trp and Met-Phe are the most common substrates) [100].

Extensive studies of N^α^-terminal acetylation in yeast and mammalian systems [101] have shown that both yeast and mammalian Nats are associated with ribosomes [102, 103]. Cross-linking experiments in *S. cerevisiae* revealed NatA association with the large ribosomal subunit via a NatA auxiliary subunit (Nat1p) that anchored the catalytic Nat subunit to the 60S ribosome [102, 103]. In addition, NatB and NatC were found to be associated with translating ribosomes [103]. NatA was shown to interact with ribosomal proteins L25 and L35 and, therefore, also uses the “universal” L25 (L23) docking site in the vicinity of the ribosome tunnel exit (as does NAC, see above) [102, 103]. L35 is also expected to be in close proximity to the tunnel exit [17, 18, 22].

Nats are expected to act on nascent chains almost immediately as they emerge from the tunnel exit (nascent chains of ∼41–47 amino acids in length have been shown to be acetylated [99–101]). However, yeast Nat1p was shown to require longer nascent polypeptides for interaction than NAC and/or Ssbs [102]. Interplay between various proteins and protein complexes docking near the tunnel exit and the hierarchy of their binding to the ribosome and/or the nascent chains is currently unknown and represents an interesting and challenging question to be answered.

Despite its prevalence, the exact biological role of N-terminal acetylation is unknown [99–101], and it is likely to have pleiotropic effects. Nat mutants in yeast exhibit diverse phenotypes, which include, but are not limited to, slow growth, temperature sensitivity, osmotic sensitivity, deficiency in utilization of nonfermentable carbon sources, inability to form functional actin cables, etc. [104]. Acetylation of yeast ribosomal proteins was recently shown to play important roles in the protein synthesis activity of ribosomes and in the maintenance of translational fidelity [104]. It was originally suggested (and widely believed) that N-terminal acetylation protects proteins from degradation. However, it was recently shown in *S. cerevisiae* that N-terminal-acetylated methionine can act as a degradation signal, targeted by the Doa10 ubiquitin ligase [105].

#### 4.5.4 N^α^-terminal myristoylation

Lipid modification of nascent chains can change protein subcellular localization, direct proteins to various cellular membranes and/or affect protein–protein interactions [106]. Myristoylation involves addition of the 14-carbon saturated fatty (myristic) acid to the N-terminal glycine of proteins [106]. Myristoylation takes place after removal of the initiator methionine from the nascent polypeptide by a methionyl aminopeptidase. This modification can be found in a variety of eukaryotic proteins of cellular and viral origin [106]. The reaction is catalyzed by myristoyl-CoA:protein *N*-myristoyl-transferase (NMT) [106]. In vertebrates, there are two NMT family members, NMT1 and NMT2 [106]. The general consensus sequence recognized by NMTs at the N terminus of a protein is Gly-X_3_-X_4_-X_5_-(Ser/Thr/Cys)_6_ [106]. It has been shown that the ∼10-kDa N-terminal domain of human NMT is both necessary and sufficient for recruitment of NMT to the ribosome [107]. However, the exact docking site of NMTs on the ribosome surface remains unknown. It is expected that, similar to other enzymes acting on elongating nascent chains, it may be positioned in the vicinity of the ribosome tunnel exit. In addition, while myristoylation was originally thought to be exclusively a co-translational protein modification [108], recent reports indicate that it can also occur post-translationally [107]. In this case, it is expected that NMT would act as a cytosolic factor without involvement of ribosome binding. Co-translational modifications of nascent chains are not limited to those that take place in the vicinity of the ribosome tunnel exit. Many downstream modification events in the life of polypeptide chains (involving longer nascent peptides) are also co-translational (Fig. 1C). As discussed below, asparagine (*N*)-linked glycosylation of polypeptides in the lumen of the ER is an example of such a downstream co-translational event [109].

## 5 Co-translational *N*-linked glycosylation

Glycosylation of asparagine residues (*N*-linked glycosylation) is one of the most ubiquitous co-translational covalent modifications of proteins in the lumen of the ER [109]. This modification involves transfer of oligosaccharides onto secretory proteins in the ER, which is catalyzed by the hetero-oligomeric complex called oligosaccharyl-transferase (OST) [109]. Mammalian OST is a membrane protein consisting of seven to eight nonidentical subunits (reviewed in reference [110]). OST uses a dolichol pyrophosphate-linked oligosaccharide precursor as the donor substrate to transfer a conserved glycan (GlcNAc2Man9Glc3) moiety to asparagine residues within consensus glycosylation sites (N-X-T/S) in the target polypeptide [109, 110]. OST is believed to be adjacent and/or associated with the translocon channel, thereby facilitating co-translational *N*-glycosylation of nascent chains [109, 110]. It is assumed that OST acts on the acceptor glycosylation sites as soon as they become available inside the ER lumen (i.e., as soon as the site is ∼65–75 amino acids away from the PTC) [109, 110]. Glycosylation increases the hydrophilicity of nascent chains and, therefore, prevents potential aggregation of polypeptides caused by exposure of hydrophobic segments that are not fully folded. After their transfer to nascent chains, *N*-glycans are subject to modification by the ER-resident enzymes glucosidase I, glucosidase II, UDP-glucose:glycoprotein glucosyltransferase and mannosidase(s) [111]. *N*-Glycan modifications appear to be critical for nascent chain interaction with the calnexin chaperone system and for overall quality control in the ER [111], which ensures production of correctly folded polypeptides and promotes ER-associated degradation (ERAD) of chains that are folded incorrectly [111]. It should be noted that all of these complex events in the ER take place after the signal peptide contained in secretory proteins is cleaved off. Cleavage of the signal peptide is also an important event during co-translational translocation of the prokaryotic secretory proteins [112].

## 6 Co-translational nascent peptide cleavage

Signal peptides play a pivotal role in directing proteins to their correct cellular and extracellular compartments (reviewed in reference [112]). Cleavage of signal peptides is (almost) an indispensable co-translational event during protein export to the periplasm and/or ER lumen in prokaryotic and eukaryotic cells, respectively (reviewed in reference [112]). Although different signal peptides do not generally share any substantial sequence homology, they do contain three conserved regions: a positively charged N-terminal region, a central hydrophobic region, and a C-terminal hydrophilic region in the vicinity of the cleavage site [112]. A family of membrane-bound signal peptidases (SPases) associated with the translocon(s) catalyzes signal peptide cleavage. Signal peptidases include type I SPases, which are found in bacteria, the ER, mitochondria, and chloroplasts, and type II SPases, which are found exclusively in bacteria [112]. Bacterial type I and type II SPases are believed to be monomeric, while eukaryotic SPases are multimeric [112]. The hydrophobic central region and the C-terminal hydrophilic region of the signal peptide are believed to span the membrane as an α-helix [112]. Cleavage is believed to occur on the periplasmic/ER lumen side of the membrane [112]. SPases are essential enzymes required for cell growth and viability [112].

Other co-translational cleavage events may also accompany maturation of nascent chains. Picornavirus polyproteins are well known to be processed through a cascade of co-translational and post-translational cleavage reactions [113]. Picornaviruses in particular employ an unusual mechanism allowing self-cleavage of the peptide bond between the 2A and 2B segments of the polyprotein. Specifically, the 2A segment (encoding the 2A protease) of the polyprotein adopts a conformation allowing self-cleavage of the polypeptide at the region between the 2A and 2B segments, which becomes temporally accessible for cleavage during co-translational folding of the polyprotein [113].

The examples discussed above clearly demonstrate that co-translational folding of nascent polypeptide chains is accompanied by a variety of complex events leading to protein processing and maturation, which together contribute to production of fully functional proteins. The process of co-translational protein folding itself will be briefly reviewed below.

## 7 Co-translational protein folding

Co-translational folding is assumed to be a universal feature of protein folding within cells, having been demonstrated for both complete and incomplete nascent polypeptides of prokaryotic and eukaryotic origin, including single- and multi-domain, single- and multi-subunit, cytosolic, secreted and membrane proteins [4–6]. All levels of protein organization can be achieved co-translationally [4–6]. As discussed above, α-helices [29, 30, 53, 59, 60] (but apparently not β-structures due to the restrictions of the geometry of the ribosome tunnel [23]) seem to form immediately at the PTC. Super-secondary structures, domains, and complete tertiary and quaternary structures are formed outside of the tunnel [4–6]. It cannot, however, be excluded that some tertiary types of interactions/structures may form at the vestibule region of the tunnel [61–63]. Hierarchical condensation of the elongating nascent polypeptide chain has been considered to be the most likely mechanism that governs folding and assembly of native proteins during synthesis in vivo [4–6]. Both steady-state and kinetic/time-resolved experiments have been extremely helpful in understanding the mechanism and pathway of protein folding in vitro (in a test tube); however, most of the evidence supporting co-translational folding has come from steady-state experiments (reviewed in reference [4]). In their snapshots of “frozen” co-translational intermediates attached to ribosomes, these studies revealed compactly folded nascent chain fragments ranging in their level of structural organization from secondary structure elements to domains and/or correctly folded full-length proteins [4–6]. There remains, however, limited understanding of how rearrangement of various structures during synthesis and co-translational folding of proteins takes place and leads to the native state. For example, it is not clear whether helices formed inside the ribosomal tunnel may undergo conversion into other structures. Interestingly, peptides with identical sequences can be found in proteins within both α- and β-structural regions [114]. It is apparent that secondary structure formation in proteins depends on the local environment [2, 3, 30, 53, 59, 60]. The tunnel was suggested to favor formation and stabilization of α-helices [30, 39, 53, 59, 60]. Therefore, it has been proposed that some α-helices formed inside the tunnel may be destabilized by local interactions as they emerge from the tunnel, resulting in their rearrangement into β- and/or other structures [58, 115]. This transition may be facilitated by ribosome pausing (see below), ribosome-associated chaperones, and/or perhaps even the ribosome itself [4, 115–121]. Interestingly, both the large (50S) ribosomal subunit and the 23S rRNA have been shown to have chaperone-like activity, in that they facilitate folding of some proteins from their unfolded states [116–119]. Specifically, domain V of the *E. coli* 23S rRNA was suggested to play an active role in this process [118]. This domain of the 23S rRNA contributes to formation of the PTC and the tunnel entrance [21, 22]. It is therefore possible that certain parts of the tunnel (perhaps the entrance and vestibule regions as well as the adjacent surface of the ribosome) might actively facilitate folding of some proteins.

Thus, a nascent polypeptide starts to fold immediately after its synthesis at the PTC and then continues (in a vectorial manner) to assume more complex structures as soon as it exits the tunnel. As discussed above, a nascent polypeptide may be subject to multiple interactions and modifications during co-translational folding, but the protein nevertheless successfully reaches its native state. This raises the intriguing question of whether a unique co-translational protein folding pathway exists. Obviously, co-translational protein folding has a number of unique and characteristic features [4–6]. Most importantly, it is a vectorial process that is coupled to translation elongation [4–6]. Many recent reports [120–123] provide overwhelming support for the 20 year-old hypothesis stating that translation elongation rates may provide an active conjunction of synthesis and folding of proteins by allowing temporal separation of sequential folding events [115, 124]. It should be noted, however, that non-uniform elongation was also suggested to modulate mRNA stability [125] and control protein expression levels [126, 127].

### 7.1 Non-uniformity of translation rates, synonymous codon usage and temporal separation of co-translational folding events

Rates of nascent chain elongation are not uniform (reviewed in reference [4]). Translation was shown to proceed more rapidly at some mRNA regions than others (reviewed in reference [4]). The initial evidence demonstrating discontinuous elongation rates came from the seminal publications by Morris and colleagues, who showed that eukaryotic (globin) and prokaryotic (MS2 phage coat) proteins were translated at rates that were not constant [128, 129]. Subsequently, this phenomenon has been demonstrated for numerous proteins from various organisms [4, 130]. Chaney and Morris first proposed mRNA secondary structure as the primary cause of non-uniform translation [129]. They attributed translation pause sites observed during the synthesis of the MS2 phage coat protein to complex MS2 RNA secondary structure (i.e., hairpin mRNA regions causing movement of the ribosome to slow down [129]). However, it became clear from later studies that, in most of cases, mRNA structure is not the primary cause of translation non-uniformity Rather, it was found that the distribution of codons with different usage frequencies along an mRNA specifies local rates of translation [4]. This was subsequently shown to also explain translation pausing in the case of the MS2 coat protein [131]. Careful analysis of the impact of RNA structure has demonstrated that after translational initiation (particularly in eukaryotes), the ribosome can, in most cases, locally destabilize secondary structures and move along the message without any significant delays [132]. On the other hand, very complex secondary structures (e.g., those involving pseudoknots) can stall elongating ribosomes, resulting in frameshifts or significant reductions in protein expression levels [133, 134]. Neverthless, non-uniform rates of translation are generally accepted to originate primarily from non-uniform usage of synonymous codons along mRNAs.

Notably, the genetic code is degenerate and synonymous codons are utilized with different frequencies and a strong codon bias exists within any given organism [135]. Moreover, the abundance of cognate tRNAs is directly proportional to the frequency of codon usage in a given organism [136]. This correlation implies that frequently used codons will be translated more rapidly than infrequently used codons and vice versa. Therefore, a cluster of rare codons is expected to slow down ribosome movement on the mRNA and lead to a translational pause [4].

It has been proposed that modulation of translation elongation rates along an mRNA might serve to fine tune the co-translational folding of the nascent polypeptide chain, ensuring its high accuracy and efficiency [115, 124]. Translational pauses might thus serve as interpunctuations temporally separating co-translational folding events [4, 115, 120–124]. We have recently reviewed [4] evidence in support of this hypothesis, which in the past 5 years has attracted an increasing number of proponents [4, 115, 120–124]. Notably, synonymous codons are distributed along mRNAs non-randomly and have been identified in clusters at certain regions that are believed to be crucial for protein folding [4, 115, 120–124]. In a family of structurally homologous proteins, this distribution of rare codons along mRNAs appeared to be well conserved despite differences in codon biases between the organisms [4, 137].This supports the possibility that the kinetics of protein translation may play a substantial role in the in vivo folding process and serve as a kinetic guide for co-translational folding [4]. Translation pauses were proposed to separate synthesis of different secondary structures [115], in particular, providing a time delay that might allow and facilitate conversion of α-helices, originally formed inside the ribosomal tunnel, into other structures, and further also provide a time delay necessary for independent folding of larger folding units such as domains (reviewed in reference [4]). The importance of synonymous codon usage and the kinetics of protein translation have been demonstrated in experiments showing that both artificial synonymous codon substitutions and naturally occurring silent single nucleotide polymorphisms can alter protein folding and conformation [4, 138, 139]. In addition, when constructs for heterologous protein expression were adjusted to utilize codon frequencies along the mRNA, similar to that observed naturally, the resulting amount of correctly folded soluble proteins increased [140, 141]. Variations in the usage of synonymous codons and in the abundance of corresponding tRNAs are also observed between different cell and tissue types in multicellular organisms [142]. This presents the possibility of cell/tissue type-specific adaptation of codon usage along an mRNA to achieve translation kinetics that are optimal for correct protein expression and folding in different cell/tissue types [142]. Interestingly, a bias in the abundance of different tRNAs was also observed in a unicellular organism (*E. coli*) at different growth rates [143]. This was proposed to reflect a mechanism for ensuring efficient and differential expression of mRNAs utilizing different synonymous codons at different stages of cellular growth [143]. Such adaptation may also contribute to ensuring correct protein folding.

The importance of synonymous codon usage and translation pausing is not limited to temporal separation of folding events. Ribosome pausing may also facilitate co-translational binding of co-factors (such as, e.g., heme) [130, 144, 145] and facilitate integration of transmembrane segments of membrane proteins into membranes [144, 146]. In addition, as mentioned above, non-uniform codon clustering may help protect mRNAs from degradation [125].

Taken together, these observations strongly suggest that mRNAs contain an additional layer of information beyond their amino acid sequence [4] and that the genetic code might not be as redundant as originally thought. This is supported by the finding that not all synonymous codon substitutions are neutral and silent [147].

## 8 Co-translational protein degradation

Cells have numerous sophisticated mechanisms to control the quality of newly synthesized proteins, many of which operate co-translationally [111, 148]. One such mechanism is co-translational protein degradation [7, 8]. As mentioned above, co-translational N-terminal acetylation can mark proteins for degradation [105]. Nascent chains can also undergo co-translational ubiquitination and subsequent degradation by the proteasome [7, 8]. It has been estimated that 20–55% of newly synthesized nascent polypeptide chains bearing an N-terminal degradation signal (“N-degron”) are degraded co-translationally [8]. The high frequency (∼55%) of co-translational degradation reported for some polypeptides might have resulted, at least in part, from experimental conditions such as use of artificial reporter constructs (heterologous fusion proteins) and cells (*S. cerevisiae*) overexpressing Ubr1p, the E3 ubiquitin ligase [8]. E3 ubiquitin ligases are primarily responsible for recognition of N-degron [149]. Interestingly, it was recently shown that another yeast E3 ubiquitin ligase, Ltn1, which acts in the quality control of proteins produced from non-stop mRNA (see above), may associate with 60S ribosomes [150]. It is reasonable to suggest that under normal circumstances only a small fraction of nascent chains (<10%) are subjects of co-translational degradation [7]. Overall, many aspects of the mechanism(s) that couple protein synthesis and degradation remain unknown. The “translasome”, a protein complex containing proteasomes and many components of the translation machinery (including ribosomal proteins and initiation and elongation factors), was recently discovered and suggested to actively couple protein synthesis, quality control and degradation [151].

The occurrence of co-translational degradation of nascent chains indicates that non-productive intermediates arise during co-translational folding. Therefore, productive co-translational pathway(s) may be in kinetic competition with pathways that target nascent chains for degradation. This suggests that co-translational folding intermediates are populated and that there may thus be a number of co-translational folding pathways leading to the native state. It is possible that modulation of the kinetics of translation by synonymous codon usage may help nascent chains follow the most productive pathway.

## 9 Conclusions

We have provided a brief overview of the complex co-translational events accompanying biogenesis of nascent polypeptide chains (Fig. 1). We believe that future studies should focus on characterization of the dynamic nature of folding intermediates that arise along the co-translational pathway and on analysis of the mechanism(s) of their structural inter-conversions as they mature.

In addition, it should be a priority to learn more about the active role that mRNA codon usage appears to play in coordinating protein synthesis and folding and in guiding co-translational folding towards the most productive pathway. Recent evidence clearly indicates that synonymous codon substitutions are not random and are subjects to constraints [4, 147]. Moreover, synonymous codon substitutions were shown to be associated with various diseases [152]. Therefore, studies of co-translational folding and the impact of synonymous codon substitutions on this process are of immense importance for our understanding of the general mechanism of protein folding as well as the origin of many diseases. Improved understanding of these effects could ultimately impact personalized medicine and personalized drug treatment and development programs. Such knowledge could also aid in appropriate choice of synonymous codons for codon-optimized gene variants to be used in gene therapy and/or in the design of new proteins to be used in biotechnology industry.

## Abbreviations

CLIPS: chaperones linked to protein synthesis
ER: endoplasmic reticulum
MAP: methionine aminopeptidase
NAC: nascent associated complex
Nat: N^α^-terminal acetyltransferase
NMT: *N*-myristoyltransferase
OST: oligosaccharyltransferase
PDF: peptide deformylase
PPIase: peptidyl-prolyl *cis/trans* isomerase
PTC: peptidyl transferase center
RAC: ribosome-associated complex
SPase: signal peptidase
SRP: signal recognition particle
TF: trigger factor
